# Physical Activity Level, Depression, Anxiety, and Self-Perceived Health in Spanish Adults with Migraine: A Cross-Sectional Study

**DOI:** 10.3390/ijerph192113882

**Published:** 2022-10-25

**Authors:** Ángel Denche-Zamorano, Víctor Paredes-Mateos, Raquel Pastor-Cisneros, Jorge Carlos-Vivas, Nicolás Contreras-Barraza, José A. Iturra-Gonzalez, María Mendoza-Muñoz

**Affiliations:** 1 Promoting a Healthy Society Research Group (PHeSO), Faculty of Sport Sciences, University of Extremadura, 10003 Caceres, Spain; 2 Facultad de Economía y Negocios, Universidad Andres Bello, Viña del Mar 2531015, Chile; 3 Escuela de Medicina, Facultad de Ciencias Médicas, Universidad de Santiago de Chile (USACH), Santiago 9170022, Chile; 4 Research Group on Physical and Health Literacy and Health-Related Quality of Life (PHYQOL), Faculty of Sport Sciences, University of Extremadura, 10003 Caceres, Spain; 5 Departamento de Desporto e Saúde, Escola de Saúde e Desenvolvimento Humano, Universidade de Évora, 7004-516 Évora, Portugal

**Keywords:** headache, chronic migraine, preventive treatment, pain, exercise

## Abstract

Background: Of all neurological disorders, migraine is the second most prevalent in the world and the most disabling, affecting approximately 15% of the general population. It is characterized by recurrent headaches, along with other symptoms and comorbidities such as depression and anxiety, compromising the sufferer’s perception of health. Physical activity is a preventive treatment for migraine and its comorbidities. The aim is to analyze the relationship between migraine and physical activity levels (PAL) in the adult Spanish population, as well as PAL and depression, anxiety, and self-perceived health (SPH) in people with migraine. Methods: A cross-sectional study was conducted including 17,137 participants, 1972 with migraine, using data from the Spanish National Health Survey. Non-parametric statistical tests were performed: z-test for independent proportions (to analyze intergroup differences) and chi-square test (to analyze dependence between categorical variables). Results: Migraine was related to PAL (*p* < 0.001). Inactive people had a higher prevalence of migraine than active and very active people (*p* < 0.05). PAL was related to depression, anxiety, SPH, and analgesic use in people with migraine (*p* < 0.001). Inactive people had a higher prevalence of depression, anxiety, analgesic use, and negative SPH than active and very active people (*p* < 0.05). Conclusions: Increasing PA in the population could reduce the prevalence of migraine. In people with migraine, inactivity could worsen SPH and increase depressive and anxious symptoms.

## 1. Introduction

Primary headaches have a prevalence and incidence of 35% in the general population [1], which can lead to migraines, causing a high social, economic, and health system impact [2]. Migraine is a chronic disorder of the nervous system, characterized by the occurrence of nausea, vomiting, photophobia, and sonophobia, in addition to some other less frequent complaints [3,4,5]. In addition, it can cause serious disruptions in the social and working life of sufferers [6]. Despite this, it is a pathology that commonly presents difficulties in being diagnosed, delaying its treatment, or increasing the risk of being inadequately treated [7].

This pathology represents a high percentage of consultations with different health professionals, a high prescription of medication, and a high healthcare cost [8].

Migraine is affected not only by personal factors, as the environment also influences its presentation, aggravating the clinical picture [9,10,11].

The mental health consequences include a tendency towards depression [12,13,14,15], which is very common in people with migraine [16] and is characterized by pervasive feelings of sadness, anhedonia, withdrawal, worthlessness, and hopelessness [17,18]. People with depression may present with cognitive and neurovegetative symptoms, such as difficulty concentrating, memory disturbances, anorexia, and sleep disturbances [19]. The Global Burden of Disease Study 2010 [14] identified depression as the second most prevalent cause of illness-induced disability, affecting people of all ages and social statuses and being a major impact factor on social, occupational, and interpersonal functioning. The decline in health associated with depression is described as significantly greater than that associated with other chronic diseases [20].

Along with depression, anxiety is another of the most common mental pathologies in people with migraine. Anxiety is a highly prevalent psychiatric condition [21,22,23] that is spreading globally in recent years, with anticipation of becoming a widespread problem in adulthood and old age, being a major cause of access to care contributing to high societal and individual costs [23]. In people with migraine, the prevalence of anxiety is even higher and with higher costs [24,25].

There are pharmacological and non-pharmacological treatments to prevent or combat the symptoms of migraine, although it is recommended to use a combination of them, carrying out a comprehensive intervention—pharmacological, physical, and social—prolonged over time to improve its effectiveness [2,26,27].

Among non-pharmacological treatments, regular exercise is one of those commonly recommended to reduce migraine symptoms [28], as well as for the reduction of symptoms of associated mental pathologies [29,30]. Exercise is defined as planned, structured, repetitive, and purposeful physical activity in the sense that the improvement or maintenance of physical fitness is the goal [31]. Aerobic endurance training has beneficial effects on the frequency of migraine episodes and migraine intensity, as well as on the duration of attacks and patient well-being [32]. Physical activity has been consistently shown to be associated with better physical health, life satisfaction, cognitive functioning, and psychological well-being [33]. In contrast, physical inactivity is associated with the development of psychological disorders. Physical activity has been shown to be associated with a decrease in symptoms of depression, anxiety, and migraine being an important asset, and it is encouraged that public health services financially support such interdisciplinary intervention programs and educational campaigns and that headache experts as well as general practitioners incorporate them into the therapeutic plan for their patients [28,33].

The main objectives of this research were: to analyze the relationships between migraine and the physical activity level (PAL) of the Spanish population prior to the COVID-19 pandemic, as well as the relationships between PAL and the prevalence of depression, anxiety, the use of analgesics, and SPH in the migraine population. The hypotheses of this research were: migraine will be related to the level of physical activity in the Spanish adult population, and that the level of physical activity of people with migraine will be related to their SPH and the prevalence of depression, anxiety, and analgesic use.

## 2. Materials and Methods

### 2.1. Design of the Study

This research is a cross-sectional study based on the Spanish National Health Survey 2017 (ENSE 2017) adult questionnaire [34], the last health survey conducted in Spain before the COVID-19 pandemic. The ENSE is a survey conducted in Spain every five years, promoted by the Ministry of Health, Consumer Affairs and Social Welfare (MSCBS) in collaboration with the National Statistics Institute (NSI). It collects information on the health status of the population residing in Spain. For the ENSE 2017, trained and accredited interviewers conducted the surveys between October 2016 and October 2017.

### 2.2. Participants

This research had a final sample of 17,139 participants, of which 1972 reported suffering from migraine. Starting from the initial sample that made up the ENSE 2017, the sample was as follows: 23,089 participants, residents in Spain aged over 15 years; the following inclusion criteria were applied: being considered of legal age in Spain (over 18 years), being aged under 70 years (given that they were not questioned about PA in the ENSE 2017), submitting answers to items: p.25.24a (questions corresponding to their migraine status) and p.113–p.117 (questions corresponding to PA carried out by the participants); not fulfilling any of the above cases being a reason for exclusion. Therefore, the following were excluded: 578 participants (under 18 years of age), 5312 participants (over 70 years of age), 2 participants (who did not submit data on their migraine status), and 58 participants (who did not submit all the data for the PA items). For the analyses specifically dedicated to people with migraine, those who reported not having migraine were excluded (15,167 participants). Three participants were not considered in the analyses that included the depression variable (item p.25.20a) and three participants were not considered in the analyses that included the anxiety variable (item p.25.21a).

### 2.3. Procedures

The data corresponding to the variables of interest were extracted from the microdata of the ENSE 2017, provided by the MSCBS: sex (male or female), age (years), migraine (collecting the responses to item p.25.24a: Have you ever suffered from migraine? With answers: Yes, No, Don’t know, or No answer (NS/NC)), depression (collecting the answers to item p.25.20th: Have you ever suffered from depression? With answers: Yes, No or NS/NC), anxiety (collecting the answers to item p.25.21st: Have you ever suffered from chronic anxiety? With answers: Yes, No or NS/NC). Data were based on the respondent’s self-reports, following previous studies [35,36]. As well as those corresponding to PA and the state of health, the following variables were created with these:

Physical activity level (PAL): This was created from the answers given to the items corresponding to PA, taking the data from items p.113–p.117. These items referred to the frequency (p.113) and duration (p.114) with which the participants performed vigorous physical activities, the frequency (p.115) and duration (p.116) with which they performed moderate physical activities, and the frequency with which they walked at least more than 10 min at a time (p.117). This set of questions is part of the physical activity attitude questionnaire (IPAQ) in its Spanish version. With these responses, a physical activity index (PAI) was constructed, applying the following factors used by Ness [37], adapted by Denche–Zamorano [38]:

Intensity: Vigorous activities (10) and moderate activities (5);

Frequency: More than three days a week (3), two or three days a week (2), one day a week (1), and no days a week (0);

Duration: 30 min or more (1.5) and between 1 and less than 30 min (1).

The PAI could take values between 0 and 67.5, with 67.5 being the highest possible PA score. With it, together with item p.117 (days per week walking more than 10 min in a row), the following PAL were created: inactive (PAI = 0; reported walking zero days per week more than 10 min in a row), walking (PAI = 0; reported walking one or more days per week more than 10 min in a row), active (PAI between 1 and 30), and very active (PAI greater than 30) [38].

Self-perceived health (SPH): This was created from data extracted from item G.21 (In the last twelve months, would you say that your health status has been very good, good, fair, fair, poor, very poor?), grouping the responses into: positive health (good or very good health status), fair health (fair health status), and negative health (poor or very poor health status).

An age grouping was also created based on the age of the participants, Age groups: 18–34 years, 35–49 years, 50–64 years, and 65–69 years.

### 2.4. Statistical Analysis

The normality of the data presented by the variables of interest was studied using the Kolmogorov–Smirnov test. Based on the results of this test, median and interquartile range (continuous variable: age) and absolute and relative frequencies (categorical variables: migraine, depression, anxiety, SPH, and level of physical activity) were used as references to characterize the sample, as well as non-parametric statistical tests for intergroup comparative analyses and relationships between variables. The Mann–Whitney U test was used to analyze differences between median ages by sex. The Chi-square test was used to analyze dependency relationships between categorical variables: sex and migraine, PAL, depression, anxiety, and SPH; migraine and age group and PAL; PAL and depression, anxiety, and SPH. A contrast of proportions was performed to test for differences in proportions in the conditions of different categorical variables according to sex, age group, and PAL, using the paired z-test for independent proportions.

The statistical software IBM SPSS Statistics v.25 was used for all analyses, taking a significance level of less than 0.05.

## 3. Results

There was insufficient evidence to assume that the data for the different variables of interest followed a normal distribution, given the results of the Kolmogorov–Smirnov test (*p* < 0.001).

Table 1 shows the descriptive analysis of the initial sample. The median age of the participants was 47 years, with no significant differences between sexes (*p* = 0.506 in the Mann–Whitney U-test). The prevalence of migraine was higher in women than in men, with statistically significant differences in migraine prevalence between sexes (16.1% vs. 6.5%, *p* < 0.05 in z-test). Migraine and sex showed dependence relationships (X^2^ = 388.4, df = 1, *p* < 0.001 in Chi-square test). In addition, relationships were found between PAL and sex (X^2^ = 373.4, df = 1, *p* < 0.001 Chi-square test). The proportion of very active men was higher than that of women (16.6% vs. 8.0%, *p* < 0.05 in z-test).

**Figure 1:**
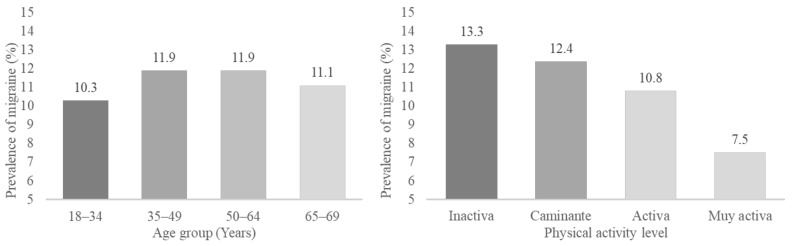
Prevalence of migraine, according to age and physical activity level.

Figure 1 and Table 2 show the prevalence of migraine according to age group and PAL. No differences in proportions were found (*p* < 0.05 in z-test), nor were dependency relationships found between migraine and age group (*p* = 0.098 in Chi-square test). The lowest prevalence of migraine was found in people with a very active PAL (7.5%), presenting a difference in proportions with the rest of the levels (*p* < 0.05 in z-test). The active group (10.8%) presented significant differences in the proportion of migraine in the z-test (*p* < 0.05) with the inactive (13.3%) and walking (12.4%) groups, with no differences between them. Dependence relationships were found between migraine and PAL (X^2^ = 49.3, df = 3, *p* < 0.001).

The characterization of the migraine population was presented in Table 3. The median age was 48 years, with no differences between men and women (*p* = 0.470 in the Mann–Whitney U-test). A prevalence of depression of 25.6% was found in people with migraine, with no differences in the proportions of people (*p* < 0.05 in the z-test), nor dependency relationships between depression and sex (X^2^ = 2.8, df = 1, *p* = 0.093). The prevalence of anxiety in this population was 25.2%, being higher in women than in men (26.9% vs. 20.7%, *p* < 0.05 in z-test). Dependence relationships were found between anxiety and sex (X^2^ = 8.0, df = 1, *p* = 0.005). Analgesic use reached a prevalence of 64%, being higher in women than in men (66.9% vs. 56.0%, *p* < 0.05 in z-test), and relationships were found between analgesic use and sex (X^2^ = 20.1, df = 1, *p* < 0.001). Only 49.8% of the migraine population reported a positive perception of their health. No differences in proportions or dependency relationships were found between SPH and sex (*p* = 0.142 in Chi-square test). A higher proportion of men were found to have a very active PAL than women (13.5% vs. 5.8%, *p* < 0.05), with no differences in the remaining levels. Dependence relationships were found between sex and PAL (X^2^ = 31.7, df = 3, *p* < 0.001).

**Figure 2:**
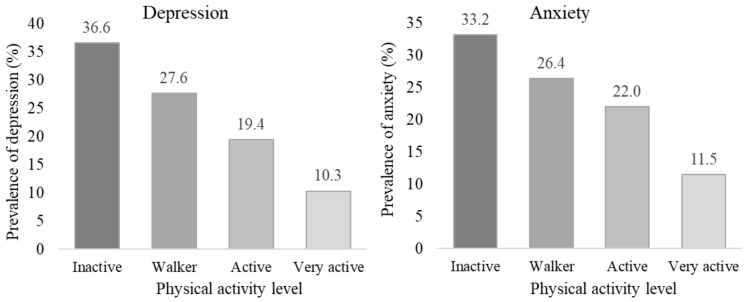
Prevalence of depression and anxiety, according to physical activity level.

Figure 2 shows the prevalence of depression and anxiety according to PAL. The lowest prevalence of depression was found in the very active population (10.3%), with differences in proportions with the rest of the level groups, and with each other (*p* < 0.05 in z-test), with a very high prevalence in the inactive population (36.6%). The lowest prevalence of anxiety was also found in the very active population (11.5%), with differences in proportions with the rest of the groups (*p* < 0.05 in z-test). Differences were found between the inactive and active groups (*p* < 0.05 in z-test), but were not found between the inactive and walker, and the walker and active groups (*p* < 0.05 in z-test).

**Figure 3:**
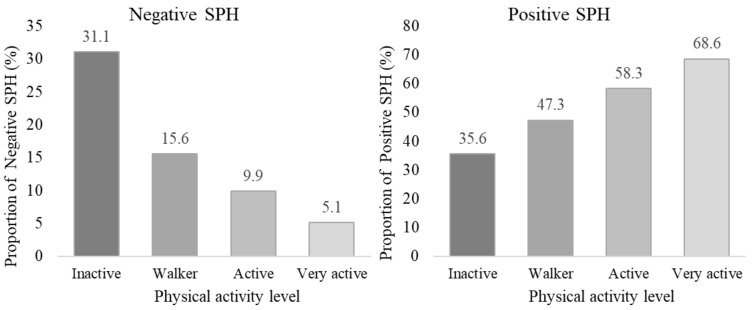
Prevalence of negative and positive self-perceived health, according to physical activity level.

As shown in Figure 3, the prevalence of negative SPH decreased from 31.1% in the inactive population to 5.1% in the very active population, and differences in proportions were found between these groups (*p* < 0.05). No differences were found between active and very active, but differences were found between the rest of the groups (Table 4).

The same differences in proportions were found in the positive SPH, from 35.6% in the inactive population to 58.3% and 68.6% in the active and very active population (*p* < 0.05 in z-test). The active (58.5%) and very active (53.2%) populations had lower prevalence of analgesic use than the inactive (70.4%) and walking (66.4%) populations. Dependency relationships were found between PAL and: depression (X^2^ = 52.2, df = 3, *p* < 0.001), anxiety (X^2^ = 30.2, df = 3, *p* < 0.001), analgesic use (X^2^ = 20.7, df = 3, *p* < 0.001), and SPH (X^2^ = 110.1, df = 6, *p* < 0.001), as can be seen in Table 4.

## 4. Discussion

The main findings of this research were: the prevalence of migraine was related to PAL, being higher in inactive people. The prevalence of depression and anxiety were found to be related to PAL in people with migraine, as well as between PAL and self-perceived health. Physically inactive people with migraine had higher prevalence of depression and anxiety, as well as a higher prevalence of negative self-perceived health than physically active people.

In the population studied, the prevalence of migraine was related to sex, being higher in women than in men (16.1% vs. 6.5%). This had already been found in other studies such as [39,40,41].

On the other hand, women presented lower proportions than men in the highest PAL, with a higher proportion of inactive women. In contrast, women presented higher proportions than men in the walker’s group. This has been found in studies such as [42,43,44,45].

The prevalence of migraine was related to PAL, being an inverse relationship. It is inverse in that as the subject has a higher level of physical activity, the prevalence of migraine decreases, with notable differences between the very active, active, walkers, and inactive groups. This difference is significant as exercise significantly reduces the migraine burden and the ability to be physically active due to the reduced impact of tension headache and neck pain [46]. Prevalence is lower in very active people and increases in the order indicated above. Physical activity plays a key role in improving and reducing migraine symptoms and associated mental health symptoms [29,30]. However, physical activity in people with migraine may be conditioned by the efficacy of the drugs used in their treatment, as some drugs cause fatigue and reduce the subjects’ exercise tolerance, encouraging physical inactivity [47,48]. Moreover, in some cases, physical activity may aggravate acute migraine symptoms, leading to a refusal of physical exercise [48].

To further improve migraine treatment through physical activity, it is advisable to use aerobic exercise, as it can decrease migraine symptoms [49]. This method was used on a sample of 36 migraineurs where moderate reductions in migraine day and migraine symptoms were observed [50].

Two comorbidities associated with migraine, depression, and anxiety are inversely influenced to a large extent by the subject’s level of physical activity.

The prevalence of depression is lower in very active people, with this figure being the lowest. As the level of physical activity decreases, the prevalence increases in the following cases: active, walkers, and inactive. This is because there is a bidirectional relationship between migraine and depression, with one disorder increasing the risk of the other and vice versa, suggesting shared biological mechanisms [51].

The same is true for anxiety in an inverse manner. The higher the level of physical activity, the lower the prevalence of anxiety in the subject, so this is largely influenced by the subject, showing that physical activity is associated with a decrease in anxiety symptoms and migraine being an important asset [28,33].

Generally, the prevalence of depression in very active people is lower in depression than the prevalence of anxiety in very active people, and in inactive people, it is higher in depression than in anxiety, according to the data corroborated by this research.

SPH is largely linked to migraine, especially physical activity. Migraine improves and worsens depending on the level of physical activity. There are two types of SPH:

Negative SPH has a high prevalence in inactive people and decreases with increasing PAL until it reaches the lowest prevalence found in very active people.

Inactive people were found to have a higher prevalence of analgesic use. This was in line with the findings of a recent article that found a lower use of analgesics, especially in women, in the German migraine population [52].

On the other hand, regarding positive SPH, the prevalence is lower in inactive subjects and increases as the PAL increases until it reaches the highest prevalence found in very active people.

SPH is impaired in people with migraines, specifically negative migraine, which increases its prevalence and is a trigger for comorbidities [53,54].

To strengthen our data and position, a study indicates that in older people between 50 and 70 years of age in Spain, the amount of PA performed has a positive effect on the perception of health, and PA performed in leisure time could be considered as an alternative to improve the quality of life of older people. An improvement in people’s SPH, in addition to being able to contribute to the reduction of health care costs associated especially with the treatment of chronic diseases, would help to improve their level of subjective well-being [55].

Migraine sufferers who are inactive have a very high prevalence of depression and anxiety, with inactivity being a crucial factor in the increase or decrease of these.

Regular exercise is one of the commonly recommended exercises to reduce migraine symptoms and is often recommended in the treatment of migraine [28], as well as for the reduction of symptoms of associated mental pathologies, such as depression and anxiety [29,30]. Therefore, the probability of having more moderate and severe symptoms of depression and anxiety compared to an active subject is much higher [56].

### 4.1. Future Considerations

It is advisable to create intervention programs that include PA as a tool to improve the symptoms of people with migraines, as well as to draw up PA guides for people with migraines. For the prevention of mental health in people with migraines, it is necessary to include PA, and it would therefore be advisable to create multidisciplinary teams to work in this area with this group. More research is needed to analyze the effects of physical activity and the prevention and treatment of migraine, and it would be recommended to develop and evaluate intervention programs to promote health through physical activity in people with migraine.

### 4.2. Limitations and Future Research

This research had the limitations inherent to cross-sectional studies, presenting difficulties in the interpretation of the associations found. It would be advisable to deepen its findings through other research that would allow causal relationships to be established. In new research, it would be advisable to include objective data on the SPH of the participants, using surveys and new references, as well as conducting studies that allow us to find more precise and concrete data on the relevance of PAL on SPH in people with migraines. The ENSE 2017 does not include medical history, nor diagnoses, and the conditions of depression, anxiety, and migraine were self-reported. This is an important limitation of this study, emphasizing the need for further research that can improve control variables. Thus, future studies should analyze the proportions of medication overuse headache in migraine patients and assess whether physical activities may or may not have an add-on effect on the reduction of migraine prevalence, since previous research has reported an association between physical activity with less analgesic use in women reporting headache [52]. Likewise, the ENSE 2017 data do not allow the prevalence of chronic migraine. In this regard, future studies should analyze and consider the prevalence and proportion of chronic migraine as well as the efficacy of preventive treatments since they could profoundly affect the prevalence of migraine and physical activities practice. This is because if migraine patients have a positive response to the preventive treatment, they may not have migraine chronification, which in turn, may lead to fewer migraine days and being able to practice more physical activities. In contrast, if migraine patients are resistant or refractory to multiple treatments, they are reluctant to perform physical activity.

## 5. Conclusions

The prevalence of migraine has a dependent relationship with physical activity level in the Spanish population. The inactive population has a higher prevalence of migraine than the active and very active populations.

In people with migraine, the prevalence of depression, anxiety, use of analgesics, and negative SPH are related to physical activity level, with active and very active people presenting a lower prevalence of these than people with a lower physical activity level. In people with migraine, it would be recommendable to maintain an active lifestyle, including moderate and/or vigorous physical activity to reduce the prevalence of depression, anxiety, analgesic use, and negative SPH.

## Figures and Tables

**Table 1:** Descriptive analysis of the general population: age, prevalence of migraine, and level of physical activity. Comparison between sexes and dependence analysis between categorical variables and sex.

Variables	Men n = 8188		Women n = 8941		Total n = 17,139		X^2^	df	*p*-Value Mann–Whitney U Test
Age									
Median (IQR)	47	(21)	47	(21)	47	(21)	-	-	0.506
Mean (SD)	46.8	(13.2)	46.9	(13.3)	46.8	(13.3)	-	-	-
Migraine	n	%	n	%	n	%	X^2^	df	*p*-value Chi-square test
Yes	532	6.5	1440	16.1 *	1972	11.5	388.4	1	<0.001
No	7666	93.5	7501	83.9 *	15,167	88.5			
PAL									
Inactive	1155	14.1	1326	14.8	2481	14.5	373.4	3	<0.001
Walker	3335	40.7	4565	51.1 *	7900	46.1			
Active	2350	28.7	2338	26.1 *	4688	27.4			
Very active	1358	16.6	712	8.0 *	2070	12.1			

X^2^ (Pearson’s Chi-square); df (degrees of freedom); n (participants); % (percentage); * (significant differences in proportions between men and women with *p*-value < 0.05 in z-test for independent proportions); PAL (physical activity level).

**Table 2:** Prevalence of migraine by age group and level of physical activity. Contrast of proportions and dependency analysis between groups and migraine status.

Migraine	18–34 Years		35–49 Years		50–64 Years		65–69 Years		Total		X^2^	df	*p*
Yes n (%)	341 a	(10.3)	734 a	(11.9)	707 a	(11.9)	190 a	(11.1)	1972	(11.5)	6.3	3	0.098
No n (%)	2956 a	(89.7)	5443	(88.1)	5247 a	(88.1)	1521	(88.9)	15,167	(88.5)			
**Migraine**	**Inactive**		**Walker**		**Active**		**Very active**		**Total**				
Yes n (%)	331 a	(13.3)	981 a	(12.4)	504 b	(10.8)	156 c	(7.5)	1972	(11.5)	49.3	3	<0.001
No n (%)	2150 a	(86.7)	6919 a	(87.6)	4184 b	(89.2)	1914 c	(92.5)	15,167	(88.5)			

X^2^ (Pearson’s Chi-square); df (degrees of freedom); n (participants); % (percentage); abc (different subscripts indicate significant differences in proportions between physical activity groups with *p*-value < 0.05 in z-test for independent proportions); *p* (*p*-value from chi-square test).

**Table 3:** Descriptive analysis of the migraine population: age, depression, anxiety, analgesics, self-perceived health, and level of physical activity. Comparison between sexes and analysis of the dependence between the categorical variables and the sex of the participants.

Variables	Men n = 532		Women n = 1440		Total n = 1972		X^2^	df	*p*-Value Mann–Whitney U Test
Age									
Median (IQR)	48.5	(19)	47	(19)	48	(20)	-	-	0.470
Mean (SD)	47.6	(12.7)	47.2	(12.6)	47.3	(12.6)	-	-	-
Depression	n	%	n	%	n	%	X^2^	df	*p*-value Chi-square test
Yes	122	22.9	383	26.7	505	25.6	2.8	1	0.093
No	410	77.1	1054	73.3	1464	74.4			
Anxiety									
Yes	110	20.7	387	26.9 *	497	25.2	8.0	1	0.005
No	387	26.9	1050	73.1 *	1969	74.8			
Analgesics									
Yes	298	56.0	964	66.9 *	1262	64.0	20.1	1	<0.001
No	234	44.0	476	33.1 *	710	36.0			
Self-perceived health									
Negative	94	17.7	220	15.3	314	15.9	3.9	2	0.142
Fair	165	31.0	510	35.4	675	34.2			
Positive	273	51.3	710	49.3	983	49.8			
PAL									
Inactive	83	15.6	248	17.2	331	16.8	31.7	3	<0.001
Walker	247	46.4	734	51.0	981	49.7			
Active	130	24.4	374	26.0	504	25.6			
Very active	72	13.5	84	5.8 *	156	7.9			

X^2^ (Pearson’s Chi-square); df (degrees of freedom); n (participants); % (percentage); * (significant differences in proportions between sex groups with *p*-value < 0.05 in z-test for independent proportions); PAL (physical activity level).

**Table 4:** Self-perceived health, analgesic intake, and prevalence of depression and anxiety in people with migraines as a function of physical activity level. Intergroup comparisons and dependency analysis between depression, anxiety, analgesic intake, self-perceived health, and level of physical activity.

Depression	Inactive		Walker		Active		Very Active		Total		X^2^	df	*p*
Yes n (%)	121 a	36.6	270 b	27.6	98 c	19.4	16 d	10.3	505	25.6	52.2	3	<0.001
No n (%)	210 a	63.4	708 b	72.4	406 c	80.6	140 d	89.7	1464	74.4			
Anxiety													
Yes n (%)	110 a	33.2	258 ab	26.4	111 b	22.0	18 c	11.5	497	25.2	30.2	3	<0.001
No n (%)	221 a	66.8	720 ab	73.6	393 b	78.0	138 c	88.5	1472	74.8			
Analgesics													
Yes n (%)	233 a	70.4	651 a	66.4	295 b	58.5	83 b	53.2	1262	64.0	20.7	3	<0.001
No n (%)	98 a	29.6	330 a	33.6	209 b	41.5	73 b	46.8	710	36.0			
Self-perceived health													
Negative n (%)	103 a	31.1	153 b	15.6	50 c	9.9	8 c	5.1	314	15.9	110.1	6	<0.001
Fair n (%)	110 a	33.2	364 a	37.1	160 a	31.7	41 a	26.3	675	34.2			
Positive n (%)	118 a	35.6	464 b	47.3	294 c	58.3	107 c	68.6	983	49.8			

X^2^ (Pearson’s Chi-square); gl (degrees of freedom); n (participants); % (percentage); abc (different subscripts indicate significant differences in proportions between physical activity groups with *p*-value < 0.05 in z-test for independent proportions).

## Data Availability

Datasets will be available under reasonable request.
